# Pediatric pycnodysostosis complicated by severe obstructive sleep apnea: a case report

**DOI:** 10.1093/omcr/omag141

**Published:** 2026-07-27

**Authors:** Islam Tarik, Farah Makhloufi, Chafiq Mahraoui, Naima El Hafidi

**Affiliations:** Department of Pediatric Infectious Diseases and Pneumo-Allergology – Pediatric I – Children’s Hospital of Rabat, University Hospital Center Ibn Sina, Rabat, Morocco; Pediatric Translational Clinical Research Unit, Biotechnology lab (MedBiotech) Bioinova Research Center, Medical and Pharmacy School, Mohammed V University in Rabat, Rabat, Morocco; Department of Pediatric Infectious Diseases and Pneumo-Allergology – Pediatric I – Children’s Hospital of Rabat, University Hospital Center Ibn Sina, Rabat, Morocco; Department of Pediatric Infectious Diseases and Pneumo-Allergology – Pediatric I – Children’s Hospital of Rabat, University Hospital Center Ibn Sina, Rabat, Morocco; Pediatric Translational Clinical Research Unit, Biotechnology lab (MedBiotech) Bioinova Research Center, Medical and Pharmacy School, Mohammed V University in Rabat, Rabat, Morocco

**Keywords:** pycnodysostosis, cathepsin K, obstructive sleep apnea, case report

## Abstract

Background: Pycnodysostosis is a rare genetic bone disorder linked to mutations in the CTSK gene, which causes osteoclast dysfunction. We report the case of a child with pycnodysostosis complicated by severe obstructive sleep apnea syndrome (OSAS). Case presentation: The patient was a 9-years-old girl born to consanguineous parents. Clinical examination revealed short stature, characteristic facial features, acromicria, and several skeletal abnormalities. X-rays revealed diffuse osteosclerosis associated with bone abnormalities characteristic of pycnodysostosis. Polysomnography confirmed severe OSA, with an apnea-hypopnea index (AHI) of 49.8 events per hour. Conclusion: This observation highlights the association between pycnodysostosis and nocturnal respiratory impairment, and underscores the importance of systematic screening for sleep-disordered breathing by polysomnography in these patients. Management is based on a multidisciplinary approach and long-term follow-up in order to optimize the functional prognosis and quality of life of patients and their families.

## Introduction

Pycnodysostosis, also known as Toulouse-Lautrec syndrome, was initially described as a condition combining osteosclerosis and short stature. Pycnodysostosis is a rare skeletal dysplasia with autosomal recessive inheritance and parental consanguinity is described in 30% of cases, with an estimated global prevalence of approximately 1 to 5 cases per million birth [1, 2]. It is caused by biallelic pathogenic variants of the CTSK gene [2]. A gene located on chromosome 1 encodes the lysosomal enzyme cathepsin K, a papain-like cysteine protease expressed mainly by osteoclasts. This enzyme plays a critical role in osteoclast driven one resorption and is essential for the degradation of collagen type I, which constitutes 95% of the organic bone matrix [3]. Deficiency in cathepsin K results in osteoclast dysfunction and lead to incomplete bone resorption, causing diffuse osteosclerosis but paradoxical bone fragility, which explains the frequency of fractures [4].

The key clinical features of pycnodysostosis include short stature and stubby hands due to distal phalenges acro-osteolysis. Craniofacial findings commonly involve underdevelopment of the maxilla and mandible, frontal and biparietal bossing, proptotic eyes, and delayed fusion of the cranial sutures. Orodental manifestations include a grooved palate, dental crowding, and hypodontia. In addition, some cases describe early eruption of primary teeth and delayed eruption of the permanent successors [5].

Beyond osteodental manifestations, otorhinolaryngological and respiratory complications are also commun in pycnodysostosis patients [6]. Craniofacial dysmorphia, narrowing of the upper airways, and certain laryngeal abnormalities predispose individuals to sleep-related breathing disorders, particularly obstructive sleep apnea syndrome (OSAS), which can be severe in children and require non-invasive ventilation [7].

We report a pediatric case complicated by severe OSAS in order to discuss diagnostic factors, treatment modalities, and the role of systematic screening for sleep-disordered breathing in these patients.

## Case presentation

A 9-year-old girl born to first-degree consanguineous parents, presented with significant short stature, associated with craniofacial dysmorphism and acromicria suggestive of pycnodysostosis and a history of recurrent lower-limb fractures. She was born at 29 weeks of gestation with a birth weight of 700 g, reflecting unexplored intrauterine growth restriction, however, prenatal growth data were not available. Her medical history was marked by three lower-limb fractures occurring at the ages of 3, 5, and 6. The fractures involved ankle fractures right and left, tibial shaft fracture following minor trauma, and were all managed by orthopedic immobilization.

**Figure 1:**
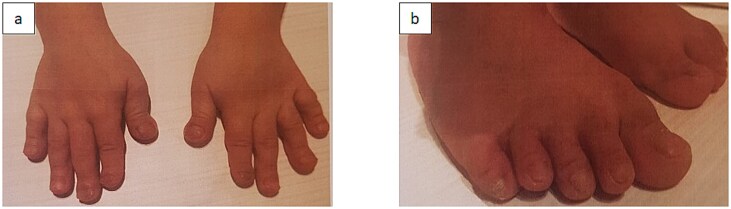
Acromicria and limb abnormalities.

Clinical examination revealed a height of 113 cm, which is 3.5 deviation below normal. The patient is overweight and has dysmorphia with low hairline and retrognathism, but no mental deficit or intellectual disability. She is intelligent, educated, and very alert. A large anterior fontanelle is also noted. In addition, she has acromicria associated with limb abnormalities (Fig. 1).

**Figure 2:**
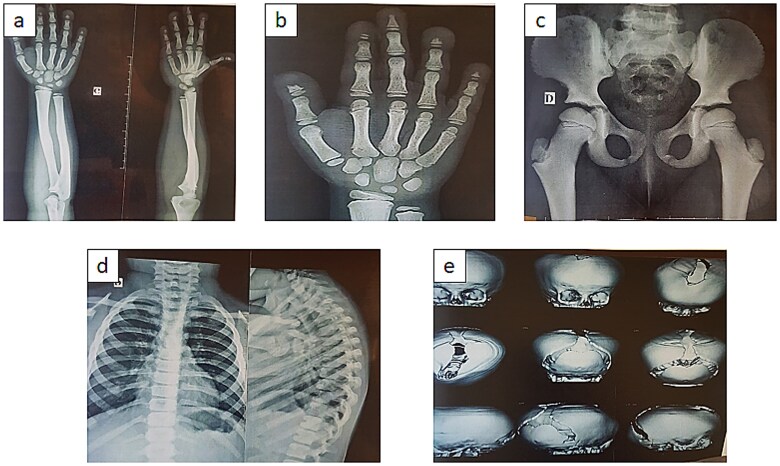
Skeletal X-rays. a. X-ray of the forearm, front and side view, showing bone hyperdensity of the radius, ulna, and metacarpals; b. X-ray of the hand showing acro-osteolysis of the distal phalanges of the 1st, 2nd, 4th, and 5th fingers with short, stocky metacarpals; c. frontal pelvic X-ray showing diffuse osteosclerosis and marked thickening of the pelvic and femoral cortex with a bilateral jagged appearance of the iliac wings; d. front and side X-ray of the spine showing scoliosis and kyphosis of the thoracic spine with vertebral bone hyperdensity; e. skull X-ray showing diffuse osteosclerosis with bone hyperdensity, widening of the sutures, delayed closure of the fontanelles, and wormian bones.

The patient did not have Insulin-like Growth Factor (IGF) deficiency (160 ng/ml) but did have low levels of creatinine (4.6 mg/l), and her calcium and phosphate blood test were normal. Skeletal X-rays revealed diffuse osteosclerosis with widening of the cranial sutures and delayed closure of the fontanelles and wormian bones, acro-osteolysis of the distal phalanges of the fingers predominantly involving the 1st, 2nd, 4th, and 5th fingers with short and stocky metacarpals. Spine radiographs revealed dorsal scoliosis with vertebral bone hyperdensity, and asymmetry of the thorax (Fig. 2).

In view of the clinical signs and radiological abnormalities, a diagnosis of pycnodysostosis was made. It was then decided to initiate growth hormone (GH) treatment at a dose of 0.030 mg/kg/day, with the aim of supporting growth and optimizing the patient’s height and weight development. Under treatment, the patient showed a marked initial response, with an acceleration in growth rate reaching 9 cm in 11 months. The treatment was well tolerated, with no notable adverse effects. However, despite the high doses administered, continued treatment proved disappointing and the improvement in growth rate was only observed during the first year. The final height stabilized at 139 cm, a difference of 16 cm from the target height.

The patient had a history of recurrent respiratory tract disease from early life, characterized by repeated episodes of nocturnal cough, rhinorrhea, sputum production, and fever, occurring approximately 7–8 times per year with asymptomatic intervals. She also had recurrent otitis, with 2–3 episodes per year. During follow-up she developed dyspnea and symptoms suggestive of sleep-disordered breathing, especially snoring, daytime sleepiness, and irritability. A polysomnography was performed, confirming severe obstructive sleep apnea syndrome, characterized by an apnea-hypopnea index (AHI) of 49.8 per hour. The patient was therefore placed on continuous positive airway pressure ventilation during the night.

## Discussion

Pycnodysostosis is a rare skeletal dysplasia, with autosomal recessive inheritance, characterized by diffuse osteosclerosis associated with bone fragility and stunted growth. Through this observation, we discuss the diagnostic elements and management issues, particularly the impact of growth hormone treatment.

The diagnosis of pycnodysostosis is based on clinical and radiological findings, namely short stature, craniofacial dysmorphism, delayed closure of the fontanelles/sutures, acromicria, diffuse osteosclerosis, acro-osteolysis of the distal phalanges, obtuse mandibular angle, and possible clavicular abnormalities [5]. In our observation, the severe short stature, craniofacial and acral stigmata, fractures, and compatible imaging support the diagnosis of pycnodysostosis. However, the lack of molecular confirmation is a significant limitation.

Several differential diagnoses were considered. Osteopetrosis was considered because of diffuse osteosclerosis and fracture susceptibility. However, the presence of distal phalangeal acro-osteolysis, acromicria, persistent open fontanelle, and the absence of reported hematological failure or neurological compression favored pycnodysostosis [4]. Analysis of the CTSK gene would confirm pycnodysostosis and rule out differential diagnoses. As part of a comprehensive management and genetic counseling approach, genetic testing should be recommended [3].

Respiratory manifestations are now recognized as a common element of pycnodysostosis, associated with midfacial hypoplasia, micrognathia, laryngotracheal abnormalities, and upper airway architecture predisposing to nocturnal collapse [7]. The French cohort studied by Bizaoui et al. highlights the frequency of ENT and respiratory complications, particularly obstructive sleep apnea (OSA), reported in a significant proportion of patients, with considerable use of non-invasive ventilation during childhood [8]. In our observation, polysomnography confirmed severe OSA (AHI = 49.8 events/hour), which led to treatment with continuous positive airway pressure.

Our case highlights the importance of systematic screening based on targeted questioning (snoring, breathing pauses, hypersomnolence or irritability, academic difficulties) and the performance of polysomnography, the gold standard test for objectively assessing and characterizing sleep-disordered breathing, and for guiding therapeutic management. In a recent Moroccan case series focused on sleep evalusation in pycnodysostosis, Bouhamid et al, reported that polysomnography identified moderate to severe OSA across their pediatric cohort (AHI up to 43.8/h) with clinically relevant nocturnel oxygen desaturation. Sleep related respiratory symptoms like snoring and breathing pause were frequent [9]. These findings support our observation that patients with pycnodysostosis should undergo polysomnography to investigate sleep related respiratory complications.

Stunted growth is one of the main signs of pycnodysososis, and somatotropic deficiency has been reported in some patients and warrants endocrinological evaluation [1]. In the cohort studied by Bizaoui et al., a proportion of patients received GH treatment with variable responses, suggesting that GH may improve growth in some patients, but without guaranteeing uniform benefits [8]. In our case, GH produced an initial acceleration in growth, followed by a result that was considered disappointing at long term. Several factors may contribute to a limited final benefit: relatively late initiation of treatment, severity of skeletal involvement, and above all potential comorbidities such as severe OSA and excess weight. These factors reinforce the idea that, in patients with pycnodysostosis, optimizing growth does not rely solely on GH, but on comprehensive management, that includes control of respiratory, nutritional, and orthopedic factors. The case series by Alsagheir et al, reported mixed outcomes after the use of recombinant human growth hormone (rhGH). Only three patients showed a positive response, whereas four has a poor response and three of them had no measurable response. These data support our observation of a limited response to GH in our pycnodysostosis patient and suggest that therapeutic effectiveness may depend on factors such as age at initiation, treatment duration, genotype differences and hormonal sensitivity, as discussed by the authors [10].

To conclude, we have presented a moroccan case of Pycnodysostosis in a 9-years old female patient, born to consanguineous parents and presented with short stature. The limitation of this report is the absence of genetic confirmation. However, the clinical and radiological concordance with reference descriptions confirms the diagnosis and reinforces the message of systematic screening for OSA in pycnodysostosis, noting that early diagnosis of this disorder is essential as bone deformity and its complications are difficult to manage.
